# Simulation-Based Training for Endoscopy Nurses Improves Device-to-Field Time: A Prospective Study

**DOI:** 10.1055/a-2901-5778

**Published:** 2026-07-13

**Authors:** Nelly Kanberg, Hadeel Atieh, Sinan Sharba, Yahya Al Hammada, Rima Tayfour, Naif Alhakmani, David James Tate, Samer Al-Dury

**Affiliations:** 1 Department of bioMedicine, Infectious Diseases Clinic 3570 University of GothenburgGothenburgSweden; 2 Department of Internal MedicineMedical City Hospital for Military and Security ServicesMuscatOman; 3 Institution of Medicine, Sahlgrenska Academy 3570 University of GothenburgGothenburgSweden; 4 Muscat Endoscopy Academy, Department of GastroenterologyMedical City Hospital for Military and Security ServicesMuscatOman; 5 Department of Gastroenterology and Hepatology 60200 University Hospital GhentGhentFlandersBelgium; 6 School of Public Health and Community Medicine, Sahlgrenska Academy 3570 University of GothenburgGothenburgSweden

**Keywords:** endoscopy nursing, simulation-based training, team-based training, workflow efficiency, device-to-field time, learning curve, therapeutic endoscopy

## Abstract

**Background** Assistant performance is important for endoscopy workflow, yet nurse training is often informal. We evaluated whether a structured simulation-and-theory program improves nurse-dependent workflow during live endoscopy.

**Methods** In this prospective parallel-group study, 20 endoscopy nurses were grouped 1:1 into Simulation + Theory or Control. Each nurse assisted 10 recorded live procedures performed by the same endoscopist (five gastroscopies and five colonoscopies; 200 nurse–procedure observations). The prespecified procedure mix included diagnostic biopsies, dilation, cold-snare polypectomy, hot-snare polypectomy, endoscopic mucosal resection, and endoscopic submucosal dissection. The primary endpoint was device-to-field time, defined at the procedure level using one predefined index request per procedure. The primary analysis was performed at the nurse level, comparing within-nurse change from baseline (cases 1–3) to posttraining (cases 4–10).

**Results** Groups performed similarly at baseline. From case 4 onward, the Simulation group showed shorter device-to-field times and maintained this advantage through case 10. In the primary nurse-level analysis, the between-group difference in change from baseline to posttraining corresponded to a time ratio of 0.80 (95% confidence interval 0.76–0.85; *p* < 0.001), indicating approximately 20% faster device readiness after structured training. Supportive sensitivity analyses showed the same directional pattern.

**Conclusions** A brief, structured simulation-and-theory program was associated with faster nurse device readiness during live endoscopy. These findings support ongoing structured nurse training as a practical strategy to improve workflow efficiency in endoscopy services.

## Introduction

Gastrointestinal (GI) endoscopy units are under sustained pressure from rising procedural demand and widening therapeutic scope, with many services still working through deferred caseloads and backlog effects. In this environment, quality and throughput hinge not only on the proceduralist but on the entire team. 1 2

Professional guidance increasingly frames endoscopy as an interdependent, competency-based team activity. European societies (ESGE/ESGENA) outline structured training pathways and assessment for core endoscopic practice, while nursing organizations emphasize defined roles, responsibilities, and progressive skills development for endoscopy nurses. 3 Yet, across systems, staffing models and training depth for assistants remain variable, and recent literature suggests substantial heterogeneity in the structure and duration of endoscopy nurse training programs internationally. 4

Simulation has emerged as a practical adjunct to traditional apprenticeship, improving procedural performance and accelerating early learning in endoscopy. 5 Randomized and controlled studies—including systematic reviews—show that simulation-based education can translate into better clinical metrics relative to no simulation exposure. However, most studies center on operators; rigorous data quantifying direct efficiency gains attributable to nurse training during live cases are limited.

Given expanding therapeutic complexity and persistent access pressures, clarifying whether targeted nurse training measurably improves workflow is a timely service question. Position statements also note the need for well-prepared nursing teams to ensure safe, efficient care as procedures become more complex—underscoring the rationale for structured assistant training beyond ad hoc, on-the-job learning. 3 4

In this study, we aimed to determine whether adding structured, simulator-based training, delivered through classroom and virtual teaching, model-based practical training, and subsequent live patient work improves real-world efficiency among endoscopy nurses across experience levels, compared with usual practice.

## Methods

### Study Design and Setting

Prospective, parallel-group evaluation of nurse assistance performance during routine GI endoscopy at a single tertiary unit. Twenty nurses were grouped 1:1 into a Simulation + Theory program (SIM) or Control (CTRL) using a pragmatic allocation process aimed at balancing overall endoscopy experience across study arms. Each nurse assisted 10 consecutive recorded live procedures (five gastroscopies and five colonoscopies; total 200 nurse–procedure observations). All procedures were performed by the same endoscopist.

### Participants

Eligible participants were active endoscopy nurses with 6 months to ≥10 years of experience. Ages ranged from 25 to 52 years, and experience ranged from 0.5 to 13 years. Experience was assessed primarily by years of work within the endoscopy unit. Prior therapeutic exposure was not available through formal individual nurse logbooks and was therefore assessed descriptively from a local staffing audit and supervisor knowledge of routine participation in therapeutic endoscopy lists. Because this variable was less precise than total years of endoscopy work, it was used to describe group comparability rather than as a formal primary stratification variable. All staff provided written consent for performance assessment and video review.

### Intervention and Comparator

The Simulation + Theory program was delivered in three sequential phases. First, participants underwent structured didactic classroom/virtual teaching covering the rationale and sequence of common diagnostic and therapeutic endoscopic workflows, including device recognition, tray organization, electrosurgery basics, communication during procedures, troubleshooting, and safety principles ( **Supplementary Table 1** ). Second, participants underwent hands-on practical training using our in-house simulation platforms, which used cow or camel tissue, including a colon simulator to train on tissue resection 6 and Muscat Endoscopy Academy Gastric Simulator, an upper GI workflow model to train on bleeding management and defect/perforation closure. 7 These sessions focused on nurse-dependent procedural tasks such as device preparation, device exchange, biopsy workflow, cold- and hot-snare setup, dilation workflow, injection setup, hemostasis readiness, generator changes, and retrieval steps. The practical component emphasized workflow rehearsal and device handling in addition to tissue-based training. The 10 sessions were progressive rather than identical, beginning with classroom-based theoretical instruction, followed by model-based rehearsal of simpler workflows, and then advancing to more complex therapeutic task sequences before transfer to live patient work ( **Supplementary Fig. 1A–D** ). Third, participants proceeded to live patient work, during which each nurse assisted 10 consecutive procedures under routine clinical conditions. CTRL continued routine departmental education and clinical duties without the structured simulation program during the study period.

### Study Timeline

The Simulation + Theory program was delivered over approximately 3–4 months in a staged format. Didactic classroom teaching and practical model-based training were conducted regularly, with practical sessions typically lasting approximately 4 h. Formal recording of live procedures began only after all participating nurses had completed the structured training phase. Outside the study intervention, nurses continued their routine clinical work and informal workplace learning as part of normal departmental practice; however, outcome measurements were restricted to the prespecified study phase after completion of the structured training program. Individual nurses completed their 10 recorded procedures over approximately 4–5 months depending on roster and case availability, while the overall live-case accrual period spanned approximately 7 months.

### Procedure Mix and Indexing

Per nurse: five gastroscopies (diagnostic with biopsies ± therapy) and five colonoscopies. The prespecified global mix included: one esophageal dilation in SIM and one in CTRL; in gastroscopy, two gastric cold-snare polypectomies ≤1 cm in CTRL and two gastric hot-snare polypectomies ~2 cm (recorded as 20–29 mm) in SIM; one esophageal variceal banding in each group; in colonoscopy, cold-snare polypectomy ≤1 cm in four of five examinations per nurse; endoscopic mucosal resection (EMR) 10–30 mm in two SIM cases and one CTRL case; and one rectosigmoid endoscopic submucosal dissection (ESD) ~4 cm in each group. EMR/ESD complexity was recorded using the SMSA level (one Level 4 and one Level 3 overall; remaining Levels 1 and 2). Procedures were indexed chronologically 1–10 per nurse to assess learning. Detailed procedure mix by group and learning window is shown in **Supplementary Table 2** .

### Case Assignment

Assignment of nurses to individual recorded live procedures was pragmatic and depended primarily on routine room allocation and roster availability for the single endoscopist performing all study procedures. For some planned therapeutic procedures, preference was given to nurses who had completed fewer recorded study procedures in order to maintain progress toward the prespecified 10-procedure target. The endoscopist did not formally allocate cases according to study group. Case assignment therefore reflected routine clinical scheduling with limited pragmatic balancing rather than strict random assignment at the procedure level.

### Outcomes and Mentor Definition

The primary endpoint was device-to-field time (DTF), defined at the procedure level using one predefined index request per procedure. DTF was measured as the interval from the endoscopist’s explicit request for the predefined index accessory to the moment that device was operable at the target. The predefined index request was selected according to procedure type and reflected the principal accessory relevant to that procedure, for example, biopsy forceps for diagnostic gastroscopy with biopsy, balloon for dilation, snare for polypectomy, injection needle or snare for EMR, and knife for ESD. Repeated requests for the same accessory within the same procedure were not included in the primary endpoint analysis.

Secondary workflow metrics were defined as follows. Exchange cycle time was the interval from removal of the previous device to the next device being ready for use at the field. Injection-ready time was the interval from request for injection to the injection device being primed and ready for use. Balloon-ready time was the interval from request for balloon dilation to the balloon system being ready for use. Hemostasis-ready time was the interval from request for a hemostatic accessory to that device being prepared and ready for use. Generator-change time was the interval from request for an electrosurgical setting change to confirmation that the required generator mode had been set. Retrieval-ready time was the interval from request for a retrieval accessory to that device being ready for use. These metrics were recorded only when applicable to the given procedure and were treated as supportive secondary outcomes. Mentor present (assistant_present = 1) referred to the presence of a supervising or additional pair of hands in the room during the procedure. Because the revised primary analysis was performed at the nurse level and the modest sample size limited stable multivariable inference, age, experience, mentor presence, and procedure-complexity variables were treated as descriptive and supportive characteristics rather than being included in the main inferential model.

### Data Collection and Timing

All cases were video-recorded with an on-screen room clock. A separate timer marked request and completion events in real time, and these logs were used together with video review to identify the timestamp of the predefined index request and the moment the device became ready for use at the field. Participating nurses were aware that workflow performance was being observed, but the predefined index event used for analysis was identified during subsequent review rather than signaled in real time. The full case-level procedural dataset is provided in **Supplementary Table 3** .

### Statistical Analysis

Continuous variables are summarized as median [range], and categorical variables as *n* / *N* (%). The primary analysis was performed at the nurse level, reflecting the level at which the intervention was delivered. For each nurse, the DFT values were summarized during a baseline period (cases 1–3) and a posttraining period (cases 4–10). Because time variables were right-skewed, analyses were performed on the log scale. For each nurse, mean log-transformed DFT was calculated for the baseline and posttraining periods, and the within-nurse change from baseline to posttraining was derived. The primary estimand was the between-group difference in this within-nurse change, comparing the Simulation group with the Control group. Effect estimates were back-transformed and presented as time ratios with 95% confidence intervals, where values below 1 indicate faster performance in the Simulation group. Supportive analyses repeated the primary analysis after excluding case 1 and after excluding advanced therapeutic procedures. Secondary workflow metrics were summarized descriptively because they were procedure-specific and not uniformly applicable across all cases. All analyses were conducted in R version 4.5.3. A two-sided *p* value < 0.05 was considered statistically significant for the primary outcome, while secondary findings were interpreted as supportive.

### Ethics

Ethical approval was obtained from the Medical City for Military and Security Services Medical Research Ethics Committee (MCMSS-MREC 043/2025). A waiver of patient consent was granted for workflow timing data, and participating staff provided written informed consent for performance assessment and video analysis.

## Results

### Participants

Twenty nurses were included (SIM *n* = 10; CTRL *n* = 10). Ages 25–52 years; and experience ranged 0.5–13 years. Prior therapeutic exposure was similar (5/10 per group). All recorded procedures were performed by the same endoscopist; there were no withdrawals or protocol deviations affecting outcome assessment ( Table 1 ).

**Table 1:** Baseline characteristics by group.

Group	Age, years (median [range])	Experience, years (median [range])	Prior therapeutic exposure, *n* /N (%)
SIM	27.0 [25.0–42.0]	1.8 [1.0–6.0]	5/10 (50%)
CTRL	30.5 [25.0–52.0]	2.5 [1.0–13.0]	5/10 (50%)

### Procedures

Each nurse assisted 10 consecutive cases (five gastroscopies, five colonoscopies), yielding 200 nurse–procedure observations. The prespecified mix was achieved: one esophageal dilation in each group; in gastroscopy, two gastric cold-snare polypectomies ≤1 cm in CTRL and two gastric hot-snare polypectomies ~2 cm (20–29 mm) in SIM; one esophageal variceal banding in each group; in colonoscopy, cold-snare polypectomy ≤1 cm in four of five examinations per nurse; EMR 10–30 mm in SIM (two cases) and CTRL (one case); and one rectosigmoid ESD ~4 cm in each group. EMR/ESD complexity (SMSA) comprised one Level 4, one Level 3, and the remainder Levels 1 and 2. Mentor present (assistant_present = 1) occurred in 60/200 cases (SIM 30; CTRL 30).

### Primary Outcome: DTF

**Fig. 1:**
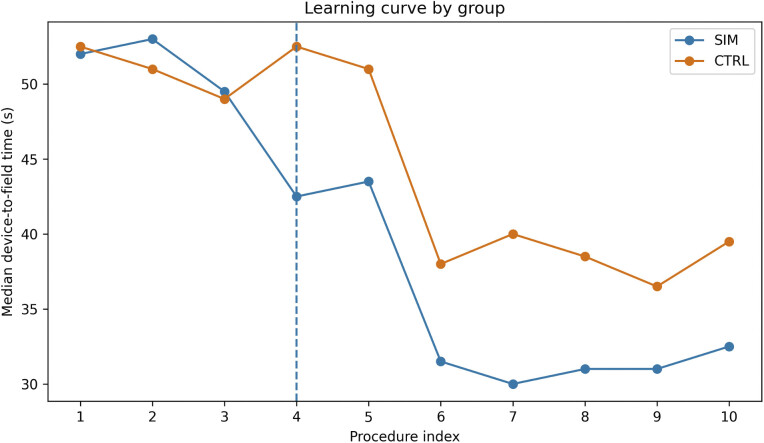
Learning curve by group. Median device-to-field time (DTF, seconds) is plotted against procedure index (1–10) for the Simulation group and the Control group. The dashed vertical line marks the transition after case 4.

At baseline (cases 1–3), groups performed similarly: SIM 52 [46–111] s versus CTRL 50 [40–108] s. From case 4 onward, the Simulation group showed shorter DFTs and maintained this advantage through case 10. In cases 4–6, median DTF was 42 [28–46] s in SIM versus 50 [35–55] s in CTRL; in cases 7–10, corresponding values were 31 [27–92] s and 39 [34–128] s, respectively ( Table 2 ). The learning-curve plot showed overlap during the baseline phase and clear separation after case 4 ( Fig. 1 ).

**Table 2:** Device-to-field time by group and learning window (in seconds).

Group	1–3	4–6	7–10
SIM	52 [46–111]	42 [28–46]	31 [27–92]
CTRL	50 [40–108]	50 [35–55]	39 [34–128]

### Primary Nurse-level Analysis

**Fig. 2:**
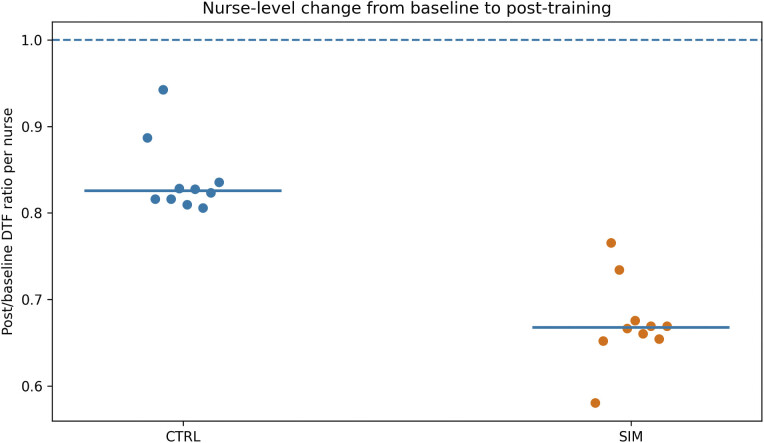
Nurse-level change from baseline to posttraining. Each point represents one nurse and shows the ratio of posttraining to baseline device-to-field time. Values below 1.0 indicate shorter device-to-field time after training; lower ratios indicate greater improvement.

In the primary nurse-level analysis, the between-group difference in change from baseline to posttraining favored the Simulation group, corresponding to a time ratio of 0.80 (95% CI 0.76–0.85; *p* < 0.001), indicating approximately 20% faster device readiness after structured training ( Table 3 , Fig. 2 ).

**Table 3:** Primary nurse-level analysis.

Analysis	Time ratio	95% CI low	95% CI high	*p* value
Primary analysis: change from baseline (cases 1–3) to posttraining (cases 4–10)	0.8	0.76	0.85	<0.001
Sensitivity analysis excluding case 1	0.79	0.72	0.87	<0.001
Sensitivity analysis excluding EMR/ESD	0.79	0.75	0.84	<0.001

### Supportive Secondary Outcomes

Supportive secondary workflow metrics showed the same directional pattern as the primary endpoint, with shorter times in the Simulation group across applicable tasks after case 4. Because these outcomes were procedure-specific and not uniformly applicable across all cases, they were treated as supportive descriptive measures and are presented in **Supplementary Table 4** .

### Sensitivity Analyses

The overall pattern was unchanged in supportive sensitivity analyses excluding case 1 and excluding advanced therapeutic procedures (EMR/ESD), with the Simulation group maintaining shorter posttraining DFTs than the Control group ( Table 3 ).

### Safety and Process Observations

No procedure-related adverse events or device malfunctions attributable to the training intervention were identified.

## Discussion

In this prospective evaluation of endoscopy nurse performance, a structured Simulation + Theory program was associated with a sustained improvement in DTF. The two groups performed similarly during the first three recorded cases and then separated from case 4 onward, with the Simulation group maintaining shorter DFTs through case 10. In the primary nurse-level analysis, the between-group difference in change from baseline to posttraining corresponded to a time ratio of 0.80, suggesting approximately 20% faster device readiness after structured training. Taken together, these findings support the view that targeted nurse training may improve key workflow components during live endoscopy.

The mechanism is plausible and consistent with the structure of the intervention. The training program focused on nurse-dependent tasks that directly influence procedural flow, including device preparation, device exchange, injection setup, hemostasis readiness, and generator adjustments. Rehearsal in a controlled environment, combined with structured teaching, may have facilitated a transition from reactive to more anticipatory assistance. Because DFT was defined using one predefined index request per procedure, the endpoint captures a focused and clinically meaningful aspect of procedural readiness rather than overall procedure duration or inspection time. 5 8

From a service perspective, these findings are relevant because delays in nurse-dependent steps can accumulate during therapeutic procedures and contribute to inefficiency even when the endoscopist’s technical performance is unchanged. 1 2 Although the present study did not measure room turnover, list overrun, or full procedure duration as primary outcomes, shorter device readiness times may translate into smoother procedural flow, particularly in units managing increasing therapeutic complexity. The observed pattern therefore points toward a potentially practical role for structured nurse training in supporting endoscopy unit efficiency.

The findings also suggest that structured rehearsal may be useful across a broad range of staff experience. The cohort included nurses with markedly different durations of endoscopy work, yet the Simulation group showed greater improvement over time than the Control group. This may indicate that focused training in high-yield workflow tasks can help standardize performance beyond what is achieved through routine experience alone. 1 3 In practical terms, ongoing and structured nurse education may be helpful not only for newer staff, but also as a way to reduce unwarranted variability within established teams.

Several features strengthen the interpretation of the study. The procedure mix was prespecified and clinically relevant, spanning both diagnostic and therapeutic endoscopy. All recorded procedures were performed by the same endoscopist, which reduced operator-related variability and allowed closer focus on assistant-dependent workflow. Timing was based on recorded video review, and the primary outcome was defined in a standardized way at the procedure level using one predefined index request per case. The consistency of the main findings across supportive sensitivity analyses further strengthens the overall signal.

The study also has important limitations. It was conducted in a single center and involved a single endoscopist, which may limit generalizability. Although 200 nurse–procedure observations were recorded, the number of nurses was modest, and the allocation process was pragmatic rather than randomized. In addition, some improvement over time may have reflected increasing familiarity within the endoscopist–assistant dyad rather than the training intervention alone. The clear separation between groups after case 4 is consistent with a training effect, but a dyad learning cannot be excluded as a contributing factor. Furthermore, the study was not designed to assess formal quality indicators, complete procedure duration, room-level throughput, or rare safety events, and the secondary workflow metrics were not uniformly applicable across all procedure types.

These considerations define the next steps for research. Future studies should examine whether similar effects are seen across multiple centers, different endoscopists, and different equipment environments. It would also be valuable to link improvements in nurse-dependent workflow to broader operational outcomes such as room efficiency, list completion, delays, and selected patient-centered or procedure-quality endpoints. Such work would help clarify how improvements in focused workflow metrics translate into endoscopy-unit performance more broadly. 4

In summary, a brief, structured simulation-and-theory program for endoscopy nurses was associated with a rapid and durable improvement in device-readiness efficiency that emerged after the initial cases and persisted thereafter. These findings support the incorporation of ongoing, structured nurse training as a practical strategy to improve workflow efficiency in endoscopy services. Whether such gains translate into broader quality and service outcomes should be examined in future studies.

**List of Abbreviations**
BRT: Balloon-ready time
CI: Confidence interval
CSP: Cold-snare polypectomy
DTF: Device-to-field time
ECT: Exchange cycle time
EGD: Esophagogastroduodenoscopy (gastroscopy)
EMR: Endoscopic mucosal resection
ESD: Endoscopic submucosal dissection
GCT: Generator-change time
GI: Gastrointestinal
HRT: Hemostasis-ready time
HSP: Hot-snare polypectomy
IRT: Injection-ready time
RRT: Retrieval-ready time
SIM: Simulation (training) group
SMSA: Size, morphology, site, access (complexity score)
TR: Time ratio
CTRL: Control group

## Supplementary Material

Ergänzendes Material Supplementary Material
