# Supplementary material for: Simulation-Based Training for Endoscopy Nurses Improves Device-to-Field Time: A Prospective Study
Source: Endosc Int Open. 2026 Jul 13;14:a29015778. doi: 10.1055/a-2901-5778 (PMC13460299; doi:10.1055/a-2901-5778)

Simulation-Based Training for Endoscopy Nurses Improves Device-to-Field Time: A Prospective Study

Supplementary Table 1. Core topics covered during classroom-based / virtual didactic teaching in the Simulation+Theory program. The classroom component preceded model-based practical training and live procedural participation and focused on nurse-dependent workflow, endoscopic device handling, electrosurgery, troubleshooting, communication, and safety.

Supplementary Table 2. Procedure mix by group and learning window. Values represent numbers of procedures classified by main procedure type across the three predefined case windows.

Supplementary Table 3. Case-level procedural dataset for all included endoscopic procedures. Each row represents one procedure and includes nurse identifier, study group, procedure index, procedure type, lesion size band, anatomical segment, mentor presence, device-to-field time, procedure-specific workflow timing metrics, and SMSA level where applicable.

Supplementary Table 4. Supportive secondary workflow metrics recorded when applicable. Results are shown descriptively as the number of applicable post-training cases and median [range] in seconds. Balloon-ready time was not available in the post-training period because both dilation procedures occurred in the baseline window.

Abbreviations: DTF, device-to-field time; ECT, exchange cycle time; IRT, injection-ready time; BRT, balloon-ready time; HRT, hemostasis-ready time; GCT, generator-change time; RRT, retrieval-ready time; TR, time ratio; CI, confidence interval; SIM, Simulation group; CTRL, Control group; EMR, endoscopic mucosal resection; ESD,

endoscopic submucosal dissection; CSP, cold-snare polypectomy; HSP, hot-snare polypectomy; SMSA, size–morphology–site–access.

Supplementary tables

Supplementary Table 1. Core topics covered during classroom-based didactic teaching in the Simulation+Theory program

No.	Topic	Focus
1	Endoscopy workflow principles (given twice)	Procedure sequence, assistant role, and anticipatory support during upper and lower GI endoscopy.
2	Accessory recognition and preparation (given twice)	Indications, setup, and handling of biopsy forceps, needles, snares, clips, dilation devices, and retrieval tools.
3	Procedure-specific assistance	Assistant workflow during biopsy, cold- and hot-snare polypectomy, dilation, EMR, and ESD.
4	Electrosurgery fundamentals	Generator modes, accessory compatibility, activation sequence, and safety checks.
5	Device exchange and procedural readiness (given twice)	Tray organization, accessory exchange, pre-procedural setup, and reduction of avoidable delay.
6	Hemostasis and troubleshooting	Preparation of hemostatic devices, response to intraprocedural bleeding, and correction of setup-related errors.
7	Communication and patient safety	Closed-loop communication, situational awareness, teamwork, and core safety principles.

Supplementary Table 2. Procedure mix by group and learning window

procedure type	CTRL 1–3	CTRL 4–6	CTRL 7–10	SIM 1–3	SIM 4–6	SIM 7–10
CSP	2	10	38	0	10	37
Dilation	1	0	0	1	0	0
EGD biopsy	27	20	0	27	20	0
EMR	0	0	1	0	0	2

ESD	0	0	1	0	0	1
HSP	0	0	0	2	0	0

Supplementary Table 3. Case-level procedural dataset

nurse id	group	procedure index	procedure type	lesion size band mm	segment	assistant present	DTF	ECT	IRT	BRT	HRT	GCT	RRT	SMSA level
SIM1	SIM	1	Dilation		Esophagus	0	111	22		117				
SIM1	SIM	2	HSP	20-29	Stomach	0	58	20				10	38	
SIM1	SIM	3	HSP	20-29	Stomach	1	50	20				10	37	
SIM1	SIM	4	EGD biopsy		Esophagus	0	43	16						
SIM1	SIM	5	EGD biopsy		Stomach	0	45	17					16	
SIM1	SIM	6	CSP	<=10	Rectum	1	28	16					29	
SIM1	SIM	7	CSP	<=10	Sigmoid	0	30	17					30	
SIM1	SIM	8	CSP	<=10	Descending	0	32	11					24	
SIM1	SIM	9	CSP	<=10	Transverse	1	33	11					24	
SIM1	SIM	10	ESD	>=30	Rectum	0	92	45	111		34	14	70	4
SIM2	SIM	1	EGD biopsy		Stomach	0	52	20					18	
SIM2	SIM	2	EGD biopsy		Stomach	0	54	21					19	
SIM2	SIM	3	EGD biopsy		Stomach	1	46	21					19	
SIM2	SIM	4	EGD biopsy		Stomach	0	42	19					18	
SIM2	SIM	5	EGD biopsy		Stomach	0	44	13					12	
SIM2	SIM	6	CSP	<=10	Rectum	1	34	12					25	
SIM2	SIM	7	CSP	<=10	Sigmoid	0	29	13					26	
SIM2	SIM	8	CSP	<=10	Descending	0	31	14					27	
SIM2	SIM	9	CSP	<=10	Transverse	1	32	14					27	
SIM2	SIM	10	EMR	20-29	Sigmoid	0	72	34	80		30	13	53	3
SIM3	SIM	1	EGD biopsy		Stomach	0	51	23					21	
SIM3	SIM	2	EGD biopsy		Stomach	0	53	17					15	
SIM3	SIM	3	EGD biopsy		Stomach	1	52	17					15	
SIM3	SIM	4	EGD biopsy		Stomach	0	41	15					14	
SIM3	SIM	5	EGD biopsy		Stomach	0	43	16					15	
SIM3	SIM	6	CSP	<=10	Rectum	1	33	15					28	
SIM3	SIM	7	CSP	<=10	Sigmoid	0	28	16					29	
SIM3	SIM	8	CSP	<=10	Descending	0	30	17					30	
SIM3	SIM	9	CSP	<=10	Transverse	1	31	10					23	

SIM3	SIM	10	EMR	20-29	Sigmoid	0	78	25	64		21	7	41	1
SIM4	SIM	1	EGD biopsy		Stomach	0	50	19					17	
SIM4	SIM	2	EGD biopsy		Stomach	0	52	20					18	
SIM4	SIM	3	EGD biopsy		Stomach	1	51	20					18	
SIM4	SIM	4	EGD biopsy		Stomach	0	40	18					17	
SIM4	SIM	5	EGD biopsy		Stomach	0	42	19					18	
SIM4	SIM	6	CSP	<=10	Rectum	1	32	11					24	
SIM4	SIM	7	CSP	<=10	Sigmoid	0	34	12					25	
SIM4	SIM	8	CSP	<=10	Descending	0	29	13					26	
SIM4	SIM	9	CSP	<=10	Transverse	1	30	13					26	
SIM4	SIM	10	CSP	<=10	Descending	0	33	15					28	
SIM5	SIM	1	EGD biopsy		Stomach	0	49	22					20	
SIM5	SIM	2	EGD biopsy		Stomach	0	51	23					21	
SIM5	SIM	3	EGD biopsy		Stomach	1	50	16					14	
SIM5	SIM	4	EGD biopsy		Stomach	0	46	14					13	
SIM5	SIM	5	EGD biopsy		Stomach	0	41	15					14	
SIM5	SIM	6	CSP	<=10	Rectum	1	31	14					27	
SIM5	SIM	7	CSP	<=10	Sigmoid	0	33	15					28	
SIM5	SIM	8	CSP	<=10	Descending	0	28	16					29	
SIM5	SIM	9	CSP	<=10	Transverse	1	29	16					29	
SIM5	SIM	10	CSP	<=10	Descending	0	32	11					24	
SIM6	SIM	1	EGD biopsy		Stomach	0	55	18					16	
SIM6	SIM	2	EGD biopsy		Stomach	0	50	19					17	
SIM6	SIM	3	EGD biopsy		Stomach	1	49	19					17	
SIM6	SIM	4	EGD biopsy		Stomach	0	45	17					16	
SIM6	SIM	5	EGD biopsy		Stomach	0	40	18					17	
SIM6	SIM	6	CSP	<=10	Rectum	1	30	17					30	
SIM6	SIM	7	CSP	<=10	Sigmoid	0	32	11					24	
SIM6	SIM	8	CSP	<=10	Descending	0	34	12					25	
SIM6	SIM	9	CSP	<=10	Transverse	1	28	12					25	
SIM6	SIM	10	CSP	<=10	Descending	0	31	14					27	
SIM7	SIM	1	EGD biopsy		Stomach	0	54	21					19	
SIM7	SIM	2	EGD biopsy		Stomach	0	49	22					20	
SIM7	SIM	3	EGD biopsy		Stomach	1	48	22					20	
SIM7	SIM	4	EGD biopsy		Stomach	0	44	13					12	
SIM7	SIM	5	EGD biopsy		Stomach	0	46	14					13	
SIM7	SIM	6	CSP	<=10	Rectum	1	29	13					26	
SIM7	SIM	7	CSP	<=10	Sigmoid	0	31	14					27	
SIM7	SIM	8	CSP	<=10	Descending	0	33	15					28	
SIM7	SIM	9	CSP	<=10	Transverse	1	27	15					28	
SIM7	SIM	10	CSP	<=10	Descending	0	30	17					30	
SIM8	SIM	1	EGD biopsy		Stomach	0	53	17					15	

SIM8	SIM	2	EGD biopsy		Stomach	0	55	18					16	
SIM8	SIM	3	EGD biopsy		Stomach	1	47	18					16	
SIM8	SIM	4	EGD biopsy		Stomach	0	43	16					15	
SIM8	SIM	5	EGD biopsy		Stomach	0	45	17					16	
SIM8	SIM	6	CSP	<=10	Rectum	1	28	16					29	
SIM8	SIM	7	CSP	<=10	Sigmoid	0	30	17					30	
SIM8	SIM	8	CSP	<=10	Descending	0	32	11					24	
SIM8	SIM	9	CSP	<=10	Transverse	1	33	11					24	
SIM8	SIM	10	CSP	<=10	Descending	0	29	13					26	
SIM9	SIM	1	EGD biopsy		Stomach	0	52	20					18	
SIM9	SIM	2	EGD biopsy		Stomach	0	54	21					19	
SIM9	SIM	3	EGD biopsy		Stomach	1	46	21					19	
SIM9	SIM	4	EGD biopsy		Stomach	0	42	19					18	
SIM9	SIM	5	EGD biopsy		Stomach	0	44	13					12	
SIM9	SIM	6	CSP	<=10	Rectum	1	34	12					25	
SIM9	SIM	7	CSP	<=10	Sigmoid	0	29	13					26	
SIM9	SIM	8	CSP	<=10	Descending	0	31	14					27	
SIM9	SIM	9	CSP	<=10	Transverse	1	32	14					27	
SIM9	SIM	10	CSP	<=10	Descending	0	28	16					29	
SIM10	SIM	1	EGD biopsy		Stomach	0	51	23					21	
SIM10	SIM	2	EGD biopsy		Stomach	0	53	17					15	
SIM10	SIM	3	EGD biopsy		Stomach	1	52	17					15	
SIM10	SIM	4	EGD biopsy		Stomach	0	41	15					14	
SIM10	SIM	5	EGD biopsy		Stomach	0	43	16					15	
SIM10	SIM	6	CSP	<=10	Rectum	1	33	15					28	
SIM10	SIM	7	CSP	<=10	Sigmoid	0	28	16					29	
SIM10	SIM	8	CSP	<=10	Descending	0	30	17					30	
SIM10	SIM	9	CSP	<=10	Transverse	1	31	10					23	
SIM10	SIM	10	CSP	<=10	Descending	0	34	12					25	
CTRL1	CTRL	1	Dilation		Esophagus	0	108	24		119				
CTRL1	CTRL	2	CSP	<=10	Stomach	0	40	18					35	
CTRL1	CTRL	3	CSP	<=10	Stomach	1	40	18					34	
CTRL1	CTRL	4	EGD biopsy		Esophagus	0	49	22						
CTRL1	CTRL	5	EGD biopsy		Stomach	0	51	23					21	
CTRL1	CTRL	6	CSP	<=10	Rectum	1	39	14					30	
CTRL1	CTRL	7	CSP	<=10	Sigmoid	0	42	15					32	
CTRL1	CTRL	8	CSP	<=10	Descending	0	37	16					33	
CTRL1	CTRL	9	CSP	<=10	Transverse	1	37	17					32	
CTRL1	CTRL	10	ESD	>=30	Sigmoid	0	128	48	116		38	17	74	2
CTRL2	CTRL	1	EGD biopsy		Stomach	0	49	22					20	
CTRL2	CTRL	2	EGD biopsy		Stomach	0	51	23					21	
CTRL2	CTRL	3	EGD biopsy		Stomach	1	50	16					14	

CTRL2	CTRL	4	EGD biopsy		Stomach	0	55	18					16	
CTRL2	CTRL	5	EGD biopsy		Stomach	0	50	19					17	
CTRL2	CTRL	6	CSP	<=10	Rectum	1	38	17					33	
CTRL2	CTRL	7	CSP	<=10	Sigmoid	0	41	18					35	
CTRL2	CTRL	8	CSP	<=10	Descending	0	36	19					36	
CTRL2	CTRL	9	CSP	<=10	Transverse	1	36	20					35	
CTRL2	CTRL	10	EMR	20-29	Ascending	0	93	31	79		26	9	50	1
CTRL3	CTRL	1	EGD biopsy		Stomach	0	55	18					16	
CTRL3	CTRL	2	EGD biopsy		Stomach	0	50	19					17	
CTRL3	CTRL	3	EGD biopsy		Stomach	1	49	19					17	
CTRL3	CTRL	4	EGD biopsy		Stomach	0	54	21					19	
CTRL3	CTRL	5	EGD biopsy		Stomach	0	49	22					20	
CTRL3	CTRL	6	CSP	<=10	Rectum	1	37	20					36	
CTRL3	CTRL	7	CSP	<=10	Sigmoid	0	40	14					31	
CTRL3	CTRL	8	CSP	<=10	Descending	0	42	15					32	
CTRL3	CTRL	9	CSP	<=10	Transverse	1	35	16					31	
CTRL3	CTRL	10	CSP	<=10	Descending	0	39	17					34	
CTRL4	CTRL	1	EGD biopsy		Stomach	0	54	21					19	
CTRL4	CTRL	2	EGD biopsy		Stomach	0	49	22					20	
CTRL4	CTRL	3	EGD biopsy		Stomach	1	48	22					20	
CTRL4	CTRL	4	EGD biopsy		Stomach	0	53	17					15	
CTRL4	CTRL	5	EGD biopsy		Stomach	0	55	18					16	
CTRL4	CTRL	6	CSP	<=10	Rectum	1	36	16					32	
CTRL4	CTRL	7	CSP	<=10	Sigmoid	0	39	17					34	
CTRL4	CTRL	8	CSP	<=10	Descending	0	41	18					35	
CTRL4	CTRL	9	CSP	<=10	Transverse	1	34	19					34	
CTRL4	CTRL	10	CSP	<=10	Descending	0	38	20					37	
CTRL5	CTRL	1	EGD biopsy		Stomach	0	53	17					15	
CTRL5	CTRL	2	EGD biopsy		Stomach	0	55	18					16	
CTRL5	CTRL	3	EGD biopsy		Stomach	1	47	18					16	
CTRL5	CTRL	4	EGD biopsy		Stomach	0	52	20					18	
CTRL5	CTRL	5	EGD biopsy		Stomach	0	54	21					19	
CTRL5	CTRL	6	CSP	<=10	Rectum	1	35	19					35	
CTRL5	CTRL	7	CSP	<=10	Sigmoid	0	38	20					37	
CTRL5	CTRL	8	CSP	<=10	Descending	0	40	14					31	
CTRL5	CTRL	9	CSP	<=10	Transverse	1	40	15					30	
CTRL5	CTRL	10	CSP	<=10	Descending	0	37	16					33	
CTRL6	CTRL	1	EGD biopsy		Stomach	0	52	20					18	
CTRL6	CTRL	2	EGD biopsy		Stomach	0	54	21					19	
CTRL6	CTRL	3	EGD biopsy		Stomach	1	46	21					19	
CTRL6	CTRL	4	EGD biopsy		Stomach	0	51	23					21	
CTRL6	CTRL	5	EGD biopsy		Stomach	0	53	17					15	

CTRL6	CTRL	6	CSP	<=10	Rectum	1	41	15					31	
CTRL6	CTRL	7	CSP	<=10	Sigmoid	0	37	16					33	
CTRL6	CTRL	8	CSP	<=10	Descending	0	39	17					34	
CTRL6	CTRL	9	CSP	<=10	Transverse	1	39	18					33	
CTRL6	CTRL	10	CSP	<=10	Descending	0	36	19					36	
CTRL7	CTRL	1	EGD biopsy		Stomach	0	51	23					21	
CTRL7	CTRL	2	EGD biopsy		Stomach	0	53	17					15	
CTRL7	CTRL	3	EGD biopsy		Stomach	1	52	17					15	
CTRL7	CTRL	4	EGD biopsy		Stomach	0	50	19					17	
CTRL7	CTRL	5	EGD biopsy		Stomach	0	52	20					18	
CTRL7	CTRL	6	CSP	<=10	Rectum	1	40	18					34	
CTRL7	CTRL	7	CSP	<=10	Sigmoid	0	36	19					36	
CTRL7	CTRL	8	CSP	<=10	Descending	0	38	20					37	
CTRL7	CTRL	9	CSP	<=10	Transverse	1	38	14					29	
CTRL7	CTRL	10	CSP	<=10	Descending	0	42	15					32	
CTRL8	CTRL	1	EGD biopsy		Stomach	0	50	19					17	
CTRL8	CTRL	2	EGD biopsy		Stomach	0	52	20					18	
CTRL8	CTRL	3	EGD biopsy		Stomach	1	51	20					18	
CTRL8	CTRL	4	EGD biopsy		Stomach	0	49	22					20	
CTRL8	CTRL	5	EGD biopsy		Stomach	0	51	23					21	
CTRL8	CTRL	6	CSP	<=10	Rectum	1	39	14					30	
CTRL8	CTRL	7	CSP	<=10	Sigmoid	0	42	15					32	
CTRL8	CTRL	8	CSP	<=10	Descending	0	37	16					33	
CTRL8	CTRL	9	CSP	<=10	Transverse	1	37	17					32	
CTRL8	CTRL	10	CSP	<=10	Descending	0	41	18					35	
CTRL9	CTRL	1	EGD biopsy		Stomach	0	49	22					20	
CTRL9	CTRL	2	EGD biopsy		Stomach	0	51	23					21	
CTRL9	CTRL	3	EGD biopsy		Stomach	1	50	16					14	
CTRL9	CTRL	4	EGD biopsy		Stomach	0	55	18					16	
CTRL9	CTRL	5	EGD biopsy		Stomach	0	50	19					17	
CTRL9	CTRL	6	CSP	<=10	Rectum	1	38	17					33	
CTRL9	CTRL	7	CSP	<=10	Sigmoid	0	41	18					35	
CTRL9	CTRL	8	CSP	<=10	Descending	0	36	19					36	
CTRL9	CTRL	9	CSP	<=10	Transverse	1	36	20					35	
CTRL9	CTRL	10	CSP	<=10	Descending	0	40	14					31	
CTRL10	CTRL	1	EGD biopsy		Stomach	0	55	18					16	
CTRL10	CTRL	2	EGD biopsy		Stomach	0	50	19					17	
CTRL10	CTRL	3	EGD biopsy		Stomach	1	49	19					17	
CTRL10	CTRL	4	EGD biopsy		Stomach	0	54	21					19	
CTRL10	CTRL	5	EGD biopsy		Stomach	0	49	22					20	
CTRL10	CTRL	6	CSP	<=10	Rectum	1	37	20					36	
CTRL10	CTRL	7	CSP	<=10	Sigmoid	0	40	14					31	

CTRL10	CTRL	8	CSP	<=10	Descending	0	42	15					32	
CTRL10	CTRL	9	CSP	<=10	Transverse	1	35	16					31	
CTRL10	CTRL	10	CSP	<=10	Descending	0	39	17					34	

Supplementary Table 4. Supportive secondary workflow metrics

Metric	Group	Applicable cases (n)	Post-training median [range], s
ECT	SIM	70	15 [10–45]
ECT	CTRL	70	18 [14–48]
IRT	SIM	3	80 [64–111]
IRT	CTRL	2	98 [79–116]
BRT	SIM	0	NA
BRT	CTRL	0	NA
HRT	SIM	3	30 [21–34]
HRT	CTRL	2	32 [26–38]
GCT	SIM	3	13 [7–14]
GCT	CTRL	2	13 [9–17]
RRT	SIM	69	26 [12–70]
RRT	CTRL	69	32 [15–74]

Supplementary Figure 1. Educational structure and simulation platform used in the Simulation + Theory program. Representative images showing (A) classroom-based didactic teaching, (B) hands-on practical workflow training with real-time timed rehearsal in the endoscopy environment. (C) The in-house designed upper and lower gastrointestinal simulation platforms. (D) Intraluminal endoscopic visualization during model-based training.

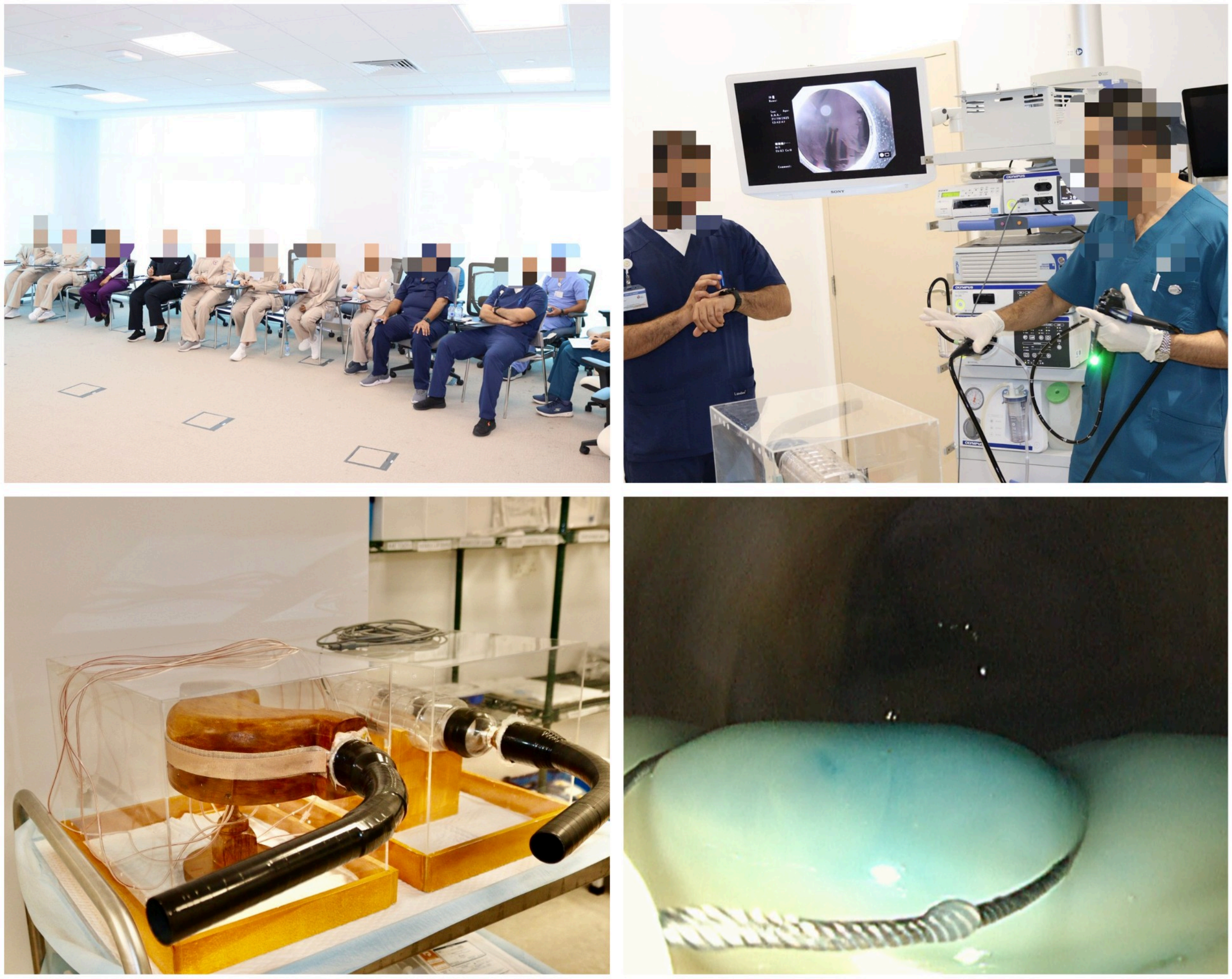

Supplement: Supplementary file 1 [file 10-1055-a-2901-5778_29021670.pdf]
